# Recent developments in using mechanistic cardiac modelling for drug safety evaluation

**DOI:** 10.1016/j.drudis.2016.02.003

**Published:** 2016-06

**Authors:** Mark R. Davies, Ken Wang, Gary R. Mirams, Antonello Caruso, Denis Noble, Antje Walz, Thierry Lavé, Franz Schuler, Thomas Singer, Liudmila Polonchuk

**Affiliations:** 1 QT-Informatics, Macclesfield, UK; 2 Roche Pharmaceutical Research and Early Development, Pharmaceutical Sciences, Roche Innovation Center Basel, Switzerland; 3 Computational Biology, Department of Computer Science, University of Oxford, OX1 3QD, UK; 4 Department of Physiology, Anatomy & Genetics, University of Oxford, OX1 3PT, UK

## Abstract

On the tenth anniversary of two key International Conference on Harmonisation (ICH) guidelines relating to cardiac proarrhythmic safety, an initiative aims to consider the implementation of a new paradigm that combines *in vitro* and *in silico* technologies to improve risk assessment. The Comprehensive *In Vitro* Proarrhythmia Assay (CiPA) initiative (co-sponsored by the Cardiac Safety Research Consortium, Health and Environmental Sciences Institute, Safety Pharmacology Society and FDA) is a bold and welcome step in using computational tools for regulatory decision making. This review compares and contrasts the state-of-the-art tools from empirical to mechanistic models of cardiac electrophysiology, and how they can and should be used in combination with experimental tests for compound decision making.

## Highlights

• Modelling and simulation can streamline decision making in drug safety testing.
• Computational cardiac electrophysiology is a mature technology with a long heritage.
• There are many challenges and opportunities in using *in silico* techniques in future.
• We discuss how models can be used at different stages of drug discovery.
• CiPA will combine screening platforms, human cell assays and *in silico* predictions.

## Teaser

In this article we present how *in silico* cardiac modelling has matured into a decision making tool in drug discovery, contrast the different approaches being proposed and show the opportunities and challenges that lie ahead for its acceptance by regulators.

## What is modelling and what are models?

Scientific models, although only reflecting simplified reality, help us to integrate our knowledge, to quantify a phenomenon and to predict outcomes; hence these models can facilitate evidence-based decision making. They can act as a repository of information for the modelled biological system allowing the viewer, researcher or modeller to understand the underlying assumptions of the model, for example which biological molecules are represented and what concentration and what association with other molecules are present. The next step is to take those static pictures and make them dynamic. For example, what happens to those biological molecules over time given a set of assumptions (model equations), initial conditions (model parameters) and concentrations of biological molecules (model variables)? This could be achieved by formulating and solving differential equations that simulate, for example, binding events, enzyme kinetics or the gating properties of cardiac ion channels.

Before we describe how models are used in drug discovery, it is worth reflecting upon the term ‘model’, which can have different meanings for different communities. At a high level, there are principally two main types of (*in silico*) models: statistical models and mechanistic models. Statistical (or empirical) models, sometimes referred to as ‘black-box models’, are built on historical data, and are ‘trained’ to imitate the trend of the data and to capture the relation between datasets. Mechanistic models are built based on our pre-knowledge of the system and the physical laws determining the system's output; they offer a descriptive advantage over the black-box approach by articulating more explicitly what is being represented by the model. Often a model that is mechanistic at one scale is black-box at another scale (e.g. we might model cell membrane potential with an electric circuit analogy but ion channel gating voltage-dependence is encoded by a statistical line of best fit equation fitted to measured data points).

Statistical models (including machine-learning approaches) often need to be trained on large datasets to increase their predictive power; a good statistical model should capture what we do not understand yet, see for example [1]. If our pre-knowledge is adequate, a mechanistic model does not need to be trained and should be predictive in new situations. However, this is infrequently the case, thus training and/or calibration datasets are often used to optimise all or a subset of the model parameters. In these cases, the lines between statistical and mechanistic models blur. Any mismatch between the reality and the output of a mechanistic model is useful, because it highlights limitations of the model that might indicate gaps in our knowledge [2]. An example of this is the prediction of the stoichiometry of the sodium/calcium exchanger when Denis Noble and Dario DiFrancesco formulated a model with dynamic concentration changes [3]. Mechanistic modelling, particularly that from a biological or physiological background, has historically included more complexity as more knowledge is learned, whereas the empiricist tries to minimise complexity. Rather than being seen as competing and isolated approaches, there is a need for a more dialectic approach with increased iteration and crosstalk between these modelling methods; we can (and should) see this as a race towards more-productive models.

## Current modelling in drug discovery and development

**Figure 1:**
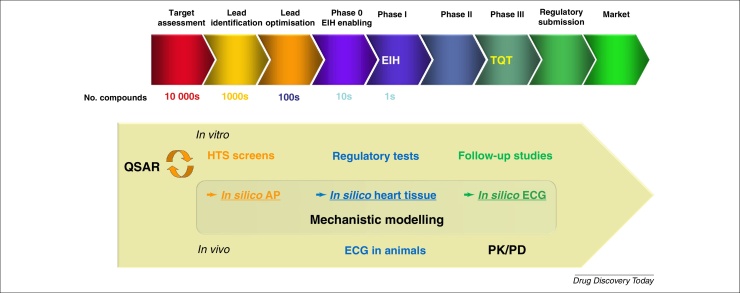
Schematic of a typical drug discovery pipeline with how and when different *in vitro*, *in vivo* and *in silico* techniques could be applied for cardiac risk assessment. In the ideal state, each of the studies should provide sufficient information to support and aid decision making fully for the ascending milestone points along the drug discovery and development pipeline. *Abbreviations*: EIH, entry into human; TQT, thorough QT study; AP, Action Potential.

Modelling and simulation (M&S) forms an integral part of the drug discovery and development process and its systematic application has been readily adopted by regulatory agencies as well as pharmaceutical research organisations 4, 5. M&S is expected to improve efficiency and productivity of drug discovery and development with its ability to test numerous scenarios *in silico* and to select those with the highest probability of success. A broad range of mathematical models are applied, with varying complexity and predictive power. The degree of model complexity is determined by the available information, the specific questions that need to be addressed and the stage of drug development [6]. We see in Fig. 1 that different modelling efforts support decision making along the drug discovery and development pipeline. Additionally, M&S is integral to decision making within the pharmaceutical industry. Translational pharmacokinetic/pharmacodynamic (PK/PD; see Glossary) modelling of efficacy and safety robustly supports a drug development programme when implemented in early-stage development 5, 7. It has the potential to project the pharmacological response in humans based on the exposure–response relationship in animal species by accounting for species differences [8]. In the early clinical development phase, it predicts the range of efficacious and tolerable target exposure and supports the selection of the most favourable dosage regimen and study design elements, such as selection of predictive PD biomarkers and PK sampling time points [5].

The impact of pharmacometrics, or modelling within clinical pharmacology, on approval and labelling decisions has greatly increased over the past decade [9]. These empirical, data-driven top-down approaches are applied to characterise the exposure–response relationship for efficacy and safety providing a quantitative assessment to guide dose selection and trial design decisions [10]. In recent years, approaches such as physiologically based pharmacokinetic (PBPK) models have been increasingly included in regulatory submissions, for example for the prediction of drug–drug interactions, drug-exposure predictions in paediatrics, in organ-impaired subjects and the effect of other patient factors [11]. Applications of PBPK specific to industry include lead optimisation and candidate selection, prediction of first-in-human PK and continue to support decision making in later phases [11]. These more mechanistic models provide a quantitative framework for prediction of systemic and tissue exposures with the distinct separation of physiology and drug-dependent information. PBPK models enable the extrapolation from *in vitro* to *in vivo* [12], from animal to human [13], from healthy volunteers to patient or special populations [11] and are applied at all stages of drug development [14]. Quantitative systems pharmacology (QSP) has emerged more recently [15] as a paradigm that combines elements of translational PK/PD and systems biology aiming to understand how the drug modulates cellular networks in space and time to predict how the pharmacological response affects the human pathophysiology [16]. This ‘middle-out’ approach (as opposed to ‘top-down’ or ‘bottom-up’), perhaps first described by Brenner *et al.* [17] and more recently by Vicini and van der Graaf [18], provides a repository of knowledge, which is powerful for mechanism-based extrapolation and presents the opportunity to predict unstudied scenarios throughout all stages of discovery and development [18]. It applies the concepts of systems engineering, systems biology and PK/PD to the study of complex biological systems through interaction between mathematical modelling and experimentation [19]. The rich heritage of mathematical cardiac electrophysiology modelling [20] makes this field one of the most mature examples of this middle-out (mechanistic) modelling approach, and as such offers a chance to define the pathway for wider uptake of QSP-type models.

Earlier in the drug discovery phase, the requirement is for an improved translation from early screening (or ideally even earlier computational chemistry) approaches to clinical cardiac outcomes, in the form of the thorough-QT (TQT) study or cardiac adverse events [21]. Key questions about compound progression are asked along this pipeline, and the value for M&S is to align and best support the decision making activities that take place. Figure 1 shows a typical drug discovery pipeline and how *in silico* cardiac modelling approaches (together with traditional experimental approaches) can support the decision points along the pathway. Ideally, more well established models such as QSAR models and simpler (e.g. classifier) models can be employed for many compounds (e.g. early chemistry-driven discovery). Later, when increasing amounts of experimental data are generated, for instance from automated patch-clamp systems, more mechanistic models can be utilised for the purpose of investigational and interpretation type work.

## Introduction to cardiac models

The groundbreaking work of Hodgkin & Huxley on squid giant axon published in 1952 [22] laid the core foundation for the mechanistic modelling of electrophysiology. This work linked the kinetics of ion channel conformation change with ion fluxes across the membrane and the change of the transmembrane potential. In 1962, Denis Noble [23] successfully extended the Hodgkin–Huxley equations to model the electrophysiology of cardiac cells. Since then, with the emerging understanding of the underlying biology – for example discovery of new ion channels [24], exchangers 25, 26 and pumps [27] – cardiac cell models have been developed further to have more-detailed representations of cellular components. These models have been brought to a relatively mature state and have been used to guide further investigation of biology and underlying mechanisms [e.g. prediction of the sodium/calcium exchanger (NCX) characteristics] [26]; to quantify certain phenomena, for example adaptation to different pacing frequency [28]; or for understanding mechanisms related to atrial fibrillation 29, 30 and hypertrophic cardiomyopathy [31]; to refine experimental protocols [32]; and more recently, prediction of drug action (reviewed in more detail in later sections). Models have been developed for different cell types: for example pacemaker cells 33, 34, atrial cells 26, 35, 36, 37, Purkinje fibres 23, 38, 39, 40 and ventricular cells 41, 42, 43, 44, 45, 46, and for different species: for example rabbit 26, 35, 47, guinea pig 42, 43, 44, human 35, 41, 45, 46, 48.

The selection of ion channels represented in cellular models can be different. This can be as a result of biological differences between cell types (e.g. funny current is mainly expressed in pacemaker cells). It can also reflect advances in the understanding of cellular biology and physiology when new ion channels are identified and characterised: the earlier models might have fewer channels, exchangers and pumps included or they have ‘lumped’ currents (e.g. a generic delayed rectifier potassium current without the separate rapid and slow components, *I*_Kr_ and *I*_Ks_) [42]. Models with ‘lumped’ currents can be inconvenient to use for drug-safety prediction, where the concept of the particular ion channel protein that is blocked by a compound needs to be linked to an individual ion current.

Ion currents can also be modelled differently. Not only can the parameter values differ (e.g. current conductance, inactivation or activation time constants) but the formulation can also differ. For example, the L-type calcium (*I*_CaL_) current can be modelled using Hodgkin–Huxley formulation (which assumes independence of different ion channel gating kinetics) [47] or a more generic Markov model [49]. One can also use Markov models with different numbers of transition states to model the same current, for example four states [50], five states [51] and six states [52] in Markov models for *I*_Kr_. An exciting new development is the derivation of Markov models from the energy landscape produced by molecular dynamics simulations [53]. At present this approach has been taken for *I*_Ks_, as more ion channel structures become known this could be an excellent way to derive Markov models.

**Figure 2:**
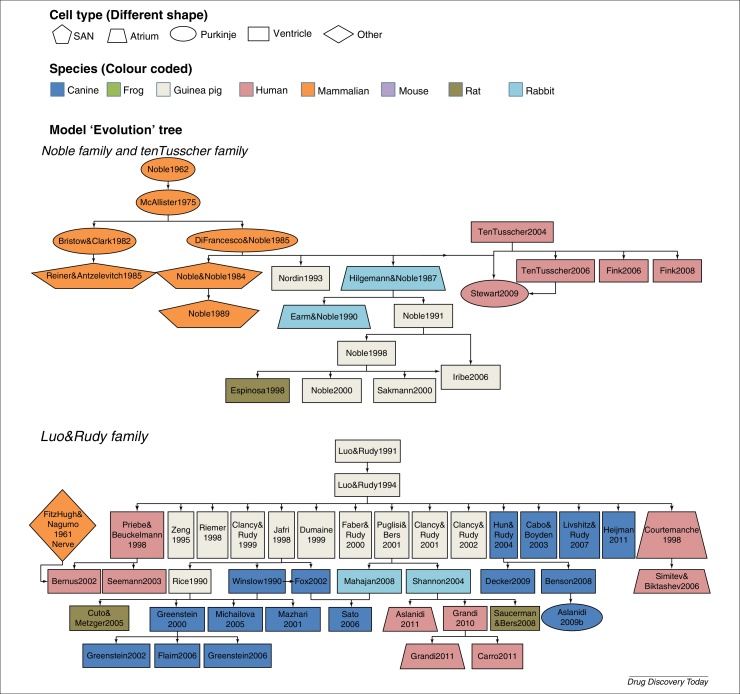
Complex heritage and interrelation between some frequently used cardiac electrophysiological cell models. Lines indicate how models inherit formulations from ‘parent’ models, whereas node colour indicates the reported species type, and shape is the reported cell type of the model. The inheritance shown in this figure was adapted, with permission, from [55].

To address the complexity of intracellular Ca^2+^ handling in the cardiac cells, cardiac cellular models can have several intracellular compartments with different calcium handling processes (e.g. Ca^2+^ release or Ca^2+^ uptake) in each compartment and Ca^2+^ diffusion between the compartments. The number of intracellular compartments can differ from one model to another from the earlier single compartment models [42] to multicompartment models 49, 54. Although cardiac cell models differ from each other in many ways, they were often built as extensions to earlier models, with some channel formulations and parameter values inherited. A 2012 review paper [55] nicely summarises this inheritance between different models, which can be viewed as a family-tree diagram in Fig. 2.

In Fig. 2, we see a complex interrelation and heritage between cardiac cell models built for different cell types or different species. One could question to what degree a model is really cell-type- and species-specific. A meta-analysis was performed [56] on two frequently used human models: ten Tusscher 2004 [41] and Iyer 2004 [57]. The authors found that although these two models were fitted to different datasets, both models were based on data obtained from multiple species: ∼50% from human, ∼25% from guinea pig and ∼25% from other species (including frog). The experimental conditions were also diverse with measurement temperatures varying from 10°C to 37°C. One needs to consider these limitations when performing a species-specific simulation or prediction. Owing to the differences in the model parameterisation and structure, even models built to simulate the same phenomenon (e.g. same cell type and same species) can behave very differently under perturbation (e.g. drug block) [58]. This is an issue (perhaps comfortingly) not solely in the domain of cardiac models. Therefore consideration for how mathematical approaches have been applied elsewhere for determining model selection could be worthwhile [59]. Awareness of the assumptions and underlying experimentation and data used for the calibration of such models is essential. One must partner that awareness with careful evaluation and validation before using these models for prediction.

Cardiac physiology is modelled not only at the single-cell level but also in multiple tissue dimensions from 1D (representing a string of cells), 2D (representing a sheet or layer of cells) through to 3D models of the whole heart and torso. To couple the single cardiac cell models together to reflect the physiology, the electrical propagation from one cell to another is modelled as the diffusion of charge throughout space, with the cardiac action potential models providing sources of charge. The equations for representing this diffusion are termed either the mono- or bi-domain equations. In the bidomain model intracellular and extracellular charges can diffuse independently, whereas the monodomain equation assumes intracellular and extracellular diffusion operate proportionally but in the same directions. The monodomain equation is a special case of the bidomain equation, and can help to reduce the computational demand of spatial models at the expense of being unable to represent changes in just extracellular currents (e.g. during defibrillation). In both cases, the complex fibre direction in the heart is represented by the diffusion of charge operating more strongly in these equations in some directions than others [60]. Although the single-cell-level is sufficient to explore the impact of ion current changes on the action potential or calcium cycling, scaling up from the single-cell- to whole-organ- and whole-body-level will be a crucial step in supporting our understanding of how the effects at the ion-channel-level translate to changes in the electrocardiogram (ECG) and phenomena such as re-entry and arrhythmia. Models at these higher scales have been reviewed elsewhere [61], in the majority of this review we focus on the cellular-level models that are becoming routinely used in drug development.

## How to handle variation in experimental data

Most cardiac models use a fixed set of parameters 33, 43, 49 – or a few distinct sets of parameters for different cell populations such as epicardial, midmyocardial and endocardial cells 62, 63 – without considering biological and experimental variability. Experimental variation has been recently demonstrated in the context of ion channel screening [64] and how to respond to this uncertainty is an important challenge for electrophysiology simulations. This variability can become important when making a prediction that accounts for intra- and inter-individual variation or expansion for an entire population where distinct subcategories of patients such as those with genetic channelopathies or underlying comorbidities (e.g. heart failure, atrial fibrillation) cause significant differences in the cellular action potentials. Population-based approaches, which can be used to fit a model to multiple datasets, have been applied to different types of modelling, for example PBPK modelling [65] and cardiac modelling 66, 67, 68. Statistical techniques, such as Bayesian inference, have also been used to parameterise models and to quantify the variability and uncertainty (with probabilities) 69, 70 which could be very beneficial for risk prediction.

## Modelling and its application to cardiac risk assessment

**Figure 3:**
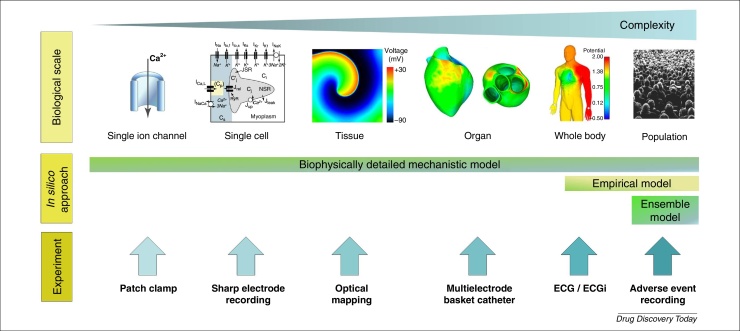
Schematic diagram showing that cardiac models (top) and corresponding experimental platforms (bottom) have been developed for use at different scales and levels of complexity. From left to right: single ion channel, single cell, 2D/3D tissue, whole organ and whole body (ECG). The biophysically detailed model has been used across scales from single ion channel to torso ECG (bottom-up modelling), whereas the empirical models tend to focus on modelling *in vivo* data such as ECG (top-down modelling). Ensemble approaches offer an opportunity to represent variants (virtual subjects) within a population. Images within the figure were adapted, with permission, from 49, 75, 81, and from StockSnap (https://stocksnap.io).

Figure 3 sets out the different scales at which measurements can be taken and *in silico* models can be used, a more detailed discussion on the opportunities for novel experimental techniques occurred recently at an interdisciplinary workshop [71]. The cardiac modelling field has advanced in the sense that *in silico* approaches and experimental measurements are now developed to the point where an assessment of the translation between different scales (e.g. between ion channel to whole cell or between *in vitro* to *in vivo*) is feasible.

At the lower scales, investigators have described the pharmacology of single ion channels and single myocardial cells with focus on mechanistic approaches. Tissue, organ and whole-body biophysical models have been employed to understand the electrophysiology of cardiac activity. At the higher scales, modelling efforts are geared towards describing ECG characteristics in the population and explaining the link to adverse cardiac events [72]. As in any application, modelling should be pragmatic and its complexity fit for purpose, often ranging from cellular models with tens of equations and parameters to simple concentration–effect relationships. It is useful to recognise that observations (that can support, for example, model calibration and validation exercises) can be made along all the different scales (e.g. from isolated ion channel recordings) through single cell recordings, whole heart wave patterns via techniques such as ECGi [73] up to consequential observations via clinical trials and the emerging discipline of real-world data via adverse event recordings and electronic health records (EHRs) [74]. As a result, academic and commercial tools, such as the preDiCT project 75, 76, the UT-Heart initiative [77] and the Cardiac Safety Simulator™ [78], that include aspects of 3D structure considerations as well as PK, single and multicellular models are being developed. These tools offer a chance to bring these multiscale measurements, after careful calibration and validation, into a single framework for decision making. Evaluating predictive power for different modelling approaches is essential for understanding how the additional complexity that is introduced into the models affects overall predictive capacity. To minimise discordance between the different scales, there is a tendency to add increasing complexity to models. This can be reflected by observing that the number of parameters in models correlates well with the CPU transistor count over time (Davies *et al*., unpublished data). It is here that an iterative approach that can simplify and add the necessary complexity should be applied; bigger is not necessarily better 79, 80.

A challenge facing drug developers in the assessment of cardiac safety is the interpretation of preclinical findings and their translation to human. The pharmaceutical industry typically evaluates a palette of *in silico*, *in vitro* and *in vivo* assays for potential cardiovascular risk before testing in human. ECG and blood pressure recordings in animal models are often used to bolster confidence in a drug candidate's *in vivo* cardiovascular effects and support decision making on the compound's suitability for progression. Various publications describe the top-down analysis of such data and PK/PD models in support of the interpretation of *in vivo* findings 82, 83, 84. To that purpose, the models attempt to elucidate and reproduce the relationship between drug exposure and observed changes in various cardiovascular endpoints (e.g. QRS, QTc, T-wave morphology, heart rate, blood pressure and contractility) 85, 86. M&S techniques enable developers and researchers to gain key insights for the assessment of a compound's therapeutic index, for instance through the extrapolation of exposures associated with the onset of cardiovascular effects or specific effect magnitudes (e.g. 10 ms QTc prolongation). These exposure–response analysis techniques have recently formed a component of data presented to support a TQT study waiver and a key part of Phase I clinical studies [87]. Often, semi-mechanistic, empirical or statistical approaches are preferred over mechanistic models, owing to the lack of, or partial understanding of, the mechanism of pharmacological action. This in turn could hinder the translatability of findings across species, and indeed discordance is sometimes observed between predictions and clinical outcomes [80].

Naturally, opportunities exist to combine bottom-up and top-down approaches to use their strengths and potentially link pharmacological effects at a cellular level to cardiac observations and clinical outcomes. A mechanistic simulation of cellular processes should allow prediction of cardiac toxicity potential in a detail unsurpassed by empirical concentration–effect relationships, particularly for compounds affecting ion channels in novel ways. Cardiac myocyte modelling holds the promise of enabling truly translational *in vitro* investigations and *in silico* extrapolations from animal to human. By contrast, the complexity of most biophysical models does not readily permit their widespread use for high-throughput risk assessment and compound prioritisation in early development. It has been suggested that these models might not yet have matured enough to add predictive power over more pragmatic approaches [79]. Further efforts can be devoted to linking cardiac myocyte model readouts for the purpose of defining the mechanisms behind the cardiac disturbance (e.g. APD_90_, triangulation, EAD propensity, upstroke velocity) to biomarkers of preclinical and clinical significance (e.g. TQT, QTc, T-wave morphology, beat-to-beat variability) 58, 88, 89, 90, 91, 92. Such attempts to link cardiac safety endpoints across multiple scales have the potential to provide a more human-relevant assessment of proarrhythmic risk earlier in drug development. Here, the mechanism-based models together with physiological experiments should be used to integrate the findings and to reduce the incidence of discordance with some drugs by providing mechanistic insights for these observations.

## Existing *in silico* evaluations for drug development decision making

The ideal scenario for drug developers and regulators is to identify that a minimal set of measurements needs to be made experimentally to predict accurately the propensity for causing arrhythmia. Preferably, these measurements would be made in early discovery and in a reproducible and high-throughput format. The outputs can then be integrated into an algorithm to provide a risk score for decision making. To date, various measurements, models and algorithms, and risk scores have been applied to this task within the pharmaceutical industry 58, 67, 79, 80, 82, 93, 94. Five of these studies use the IC_50_ score as the input values into the algorithm, whereas two of the studies 82, 94 use *in vivo* cardiovascular endpoints or gene expression signatures, respectively, as alternative strategies for the purpose of cardiac safety risk scoring.

The first study [82] demonstrated a PK/PD modelling approach for assessing cardiovascular safety when *in vivo* data are already known. In this study, the cardiovascular safety data, such as QRS complex, QTc interval, heart rate and blood pressure, could be correlated to the plasma concentrations to predict across species and to provide an estimate of therapeutic window. Whereas some ion channel data are measured in this study, the integration of this information was used only for qualitative purposes. A holistic approach was used to identify compounds with a likely cardiovascular risk [94]. This study suggests gene expression profiles can be used as a surrogate for hERG inhibition based on the premise that, although hERG inhibition is independent of structurally diverse chemicals, it is dependent on a conserved cell physiological response that can be independent of chemical diversity. They cluster gene expression fingerprints then identify those clusters where hERG channel inhibition has been previously described. Therefore *de novo* gene fingerprints that co-cluster with these hERG inhibitor enriched fingerprints are more likely to show hERG current block.

The remaining studies have all used ion channel screening data as an input with varying abstractions of statistical or mechanistically based models. Each of these studies attempts to emulate or improve on the inherent modelling performed within the mind of an experienced electrophysiologist or safety pharmacologist (i.e. *in cerebro* modelling). Variance in this strategy for predicting cardiac safety concern is also present, and referred to as a ‘matter of opinion’. A seminal study [95] was the first major attempt to classify potential risk of Torsade de Pointes (TdP) based on hERG IC_50_ data only, from 100 drugs. In their analysis the authors concluded a correlation of hERG activity with incidence of TdP, yet it is not absolute and in general a threshold of 30-fold difference between a free plasma concentration and hERG IC_50_ was proposed as sufficient to mitigate risk (except in the cases of multichannel drugs). Another study to use the Redfern categories as a surrogate of TdP risk [93] was also the first study to benchmark a mechanistic *in silico* model simulation against a drug's clinical risk. For the 31 drugs profiled, an improved prediction (i.e. Redfern categorisation) was observed when using multichannel (hERG, hNaV1.5 and hCaV1.2) data from combining literature reports with patch-clamp data versus using a ratio of hERG IC_50_ and effective free therapeutic plasma concentration (EFTPC) alone. A parallel study [67] also used a mechanistic model to integrate multichannel data to predict action potential changes from a canine cardiomyocyte assay. Here, they used an additional two channels (hKv4.3/hKChIP2.2 and hKv7.1/hminK, corresponding to the *I*_to1_ and *I*_Ks_ currents) for a set of 53 compounds that had all been profiled using the IonWorks^®^ automated patch-clamp system. This study also included an ensemble of parameter sets to represent 19 different dogs from which the model had been parameterised. A further study [96] evaluated the accuracy of an *in silico* model when presented with data from QSAR predictions or from different experimental platforms, either the PatchXpress^®^ (77 compounds) or a combination of using data from IonWorks^®^/FLIPR assays (121 compounds) for predicting QT prolongation obtained from a rabbit ventricular wedge assay.

Reemphasising the importance of considering hERG, Nav1.5 and Cav1.2 channels was a study [80] that integrated the data obtained from PatchXpress^®^ and QPatch platforms for 55 compounds divided between compounds that were either torsadogenic (32) or nontorsadogenic (23) (determined from either the Redfern study or the AZCERT database), and showed that a statistical (logistic regression) model based on multichannel data is superior to models based only on hERG data. In another study [58], ion channel screening datasets were combined (for up to five currents) from two pharmaceutical companies to evaluate the prediction of the outcome of the TQT study using simulations of APD_90_ at the EFTPC. The study importantly highlights discordance for some drugs between simulated action potential effects at EFTPC and clinical observations at EFTPC that cannot readily be explained.

The most recent study [79] shows that cardiac liability is essentially a balance between the predicted depolarising and repolarising effects. They simplified the risk assessment algorithm, similar to that described in [80], to just integrate three key cardiac ion currents: *I*_Kr_, *I*_Na_ and *I*_CaL_, as the input values for an empirical, classifier model. Other emerging areas of prediction not reliant on mechanistic modelling include machine learning approaches that attempt to use the knowledge of well-studied compounds to parameterise an empirical model that accepts ion channel data as the differentiating data between compounds (see for example: https://cardiotox-predictor.com).

## Considerations for improving the reproducibility and accuracy of cardiac risk prediction

The *in silico* approach has attracted a lot of attention and interest and has shown some promising results. However, it is vital to highlight that different models can produce very different predictions, see particularly [98] and also the Web Lab tool [97] (see https://chaste.cs.ox.ac.uk/FunctionalCuration). Understanding these differences and assessing the performance of any given model for individual context is important to avoid drawing incorrect conclusions. Modelling relies not only on diligence in building models but on a thorough and honest assessment of its limitations to avoid inappropriate predictions. In this section, we discuss some of the aspects of these models that can lead to divergence in predictions, what these divergences mean and what can be done to mitigate them.

### Selection of training and validation data

Because each study has used different input parameters (measurements and different outputs) it is not currently possible to evaluate one versus the other easily. Therefore, a carefully balanced and highly characterised evaluation set that can be routinely applied to these different systems is essential. The CiPA initiative has defined a set of 28 compounds (Table 1) that have a range of proarrhythmic potential as defined by the CredibleMeds^®^ score (http://www.crediblemeds.org). Therefore, each model system should be evaluated against a standard set of compounds to collect data across the scale continuum (Fig. 3) enabling a more thorough understanding of the translatability of these models.

Careful validation of a statistical or mechanical model's predictive performance involves testing the predictions against unseen validation data. The validation set defines the performance of a model, and should be chosen carefully to represent a context of use. In a cardiac safety situation there are various aspects to this. First, the training and validation datasets should be gathered in the same way as the data that are intended to be used for predictions when in production. Table 2 shows predictions are seen to depend strongly on: (Table 2a) the mathematical model that is used (O’Hara, ten Tusscher '06 or Grandi) when the data source (fully automated ‘Q’) is fixed; and (Table 2b) the basis of the validation dataset – using the same compounds but ion channel screening was performed using fully automated (Q) or automated and manual (M&Q) methods when the model is fixed (O’Hara model). In both cases, the concentration at which predictions are made, often unknown in early safety testing, is flexible to account for the apparent discordance between prediction and clinical observation. To provide an accurate estimate of future performance, the relevant production protocols should be used for validation data gathering. Second, the compounds that are evaluated should be representative of those that are to be assessed in future – predicting the effects of strong specific hERG blockers is necessary but not sufficient when we consider that most of the compounds that are likely to be encountered today are multichannel blockers.

In addition, it is important to know which data were used in the calibration of a statistical model (or, equally, in the selection of a mechanistic model), and these should be avoided in the validation dataset. Otherwise, a spuriously accurate validation assessment will be made for cases that are actually part of the training set; and the model will subsequently perform worse than expected on a future set of compounds. An example would be where a mechanistic model is trained, such that the model provides accurate predictions of the effect of 100 nm dofetilide on APD. Clearly, dofetilide should then be omitted from any validation dataset, because its effect will be correctly predicted by definition and hence skew the performance. Therefore users should demand a clearly defined training procedure from open- and closed-source models and software to avoid this situation.

**Figure 4:**
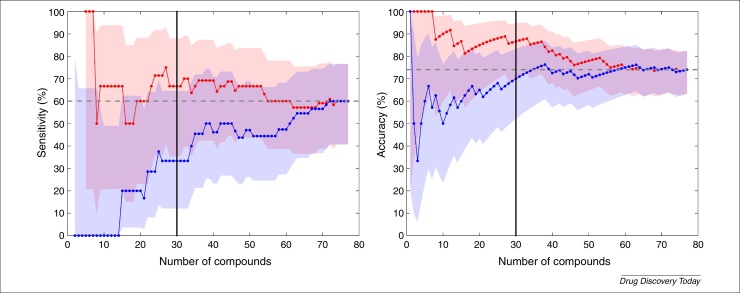
An illustration of the dependence of performance statistics, and the uncertainty in these, on the number of compounds used in a validation study. Here, we compare whether compounds caused 10% prolongation of QT interval in a rabbit left-ventricular wedge with simulations based on multiple ion channel automated PatchXPress^®^ screens. We plot the (left) sensitivity and (right) accuracy of the assay as a function of the number of compounds that are considered in the validation set (*n* = 77 available in total). The blue and red data are from the same compounds but considered in a different (randomly permuted) order, note that both measures have to start at either 0% or 100% (the first classification is right or wrong), and that the entries for *n* = 77 must be identical. 95% confidence intervals on these statistics are generated using Wilson's Score Interval, and are shown with the shaded regions. The data shown are taken, with permission, from [96] (available to download from: http://www.cs.ox.ac.uk/chaste/download.html – Jptm2013Beattie project).

There should also be confidence estimates on our assessments of predictive power. In Fig. 4 we show how an assessment of sensitivity and accuracy of a binary classifier depends on the number of compounds in a validation set, as well as the order of the compounds in this set. The real predictive power of an assay (if it were to be evaluated on an extremely large dataset) can be very different to the reported value from a single validation study. By calculating a confidence interval, in this case based on Wilson's Score Interval [99] (as previously used in this contextin [96]), we see an estimate of how much confidence we should place in the performance statistics, and see how this changes with different numbers of validation compounds. Thirty compounds might appear a reasonable number to make a judgement as to the validity of a model (black vertical line from Fig. 4); but, in this example, precisely which 30 compounds have been randomly selected can markedly impact the predictive score of the model, we see 70% versus 86.7% accuracy in the example shown.

Table 3 shows how the composition of compound sets across several *in silico* evaluation studies is variable and, therefore, the importance of a defined, well-balanced set of compounds (e.g. from the CiPA initiative) is of considerable value for making a genuine comparison possible. Although 28 compounds have appeared in more than one study, no compounds have been used in every study and only seven of those compounds have appeared in more than two studies, therefore meaning that evaluation across different studies is difficult.

Finally, the example in Fig. 4 shows how a model scores for predicting 10% prolongation of QT interval. Multiple, equally valid markers (e.g*.* 5 ms change in QT-interval) could have been chosen that would have influenced the model's performance. This becomes more pronounced when the comparator is more subjective in nature, such as a Redfern category or AZCERT ranking. These values can and do change, based on medical observation, therefore a drug with ‘no TdP risk’ cannot always be viewed as safe, see for example recent modifications of drugs such as propofol, ciprofloxacin from ‘conditional’ to ‘known TdP’ (https://www.crediblemeds.org/blog/changes-made-crediblemeds-lists1/). An opportunity therefore exists to translate cardiac models not to ‘incidence of TdP’ but rather to cardiac adverse events. This is emphasised in a study [74] that shows that, although TdP is an exemplar adverse event, a low incidence of TdP from their observational study and a lack of ICD-10 coding options mean that some important drug-induced arrhythmia events could be missed or recorded as other ventricular fibrillation events.

### Model version control

**Figure 5:**
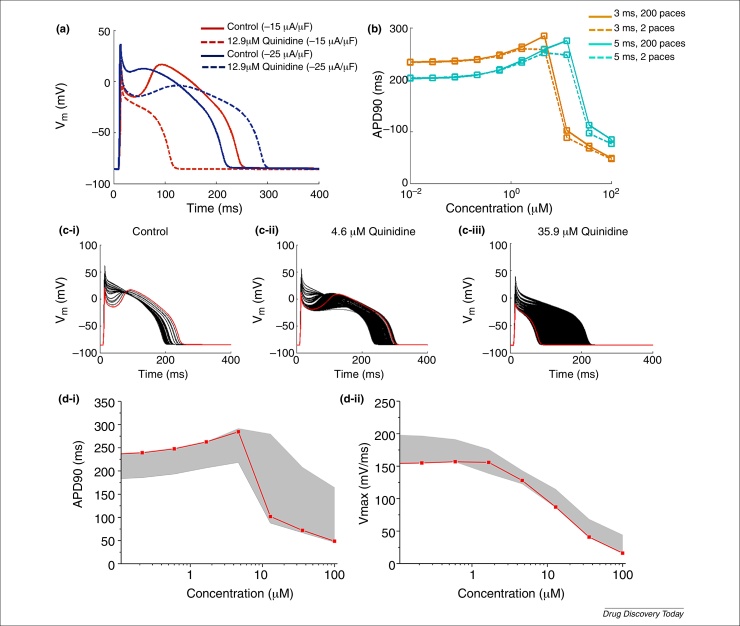
The impact of the protocol-related parameters when simulating an increasing concentration of quinidine. **(a)** The effect of stimulation pulse amplitude on AP. The solid lines show control AP and the dashed lines show the AP with 12.9 μm quinidine. The AP traces simulated with −15 μA/μF stimulation pulse and −25 μA/μF stimulation pulse, coloured in red and blue, respectively. **(b)** The APD_90_ dose–response (with ascending concentration of quinidine) simulated using 3 ms (coloured in orange) and 5 ms (coloured in turquoise) stimulation pulse and after two (shown in dashed line) or 200 (shown in solid line) paces. **(ci–iii)** Simulated AP with different concentration of quinidine when applying various pacing protocols (stimulation pulse amplitude: −15, −18, −20, −22 or −25 μA/μF; stimulation pulse duration: 3, 4 or 5 ms; number of pulses: 1–200 pulses). The red line shows the AP simulated using stimulation protocol published with the original model and after 200 pulses and black line shows the AP simulated using other variants of stimulation protocols as mentioned above. **(di,ii)** shows dose–response of the APD_90_ and maximum upstroke velocity simulated using the variants of stimulation protocol used for subfigure (ci–iii). The red line shows the simulation result obtained using the stimulation protocol published with the original model, the grey shade marks out the variability induced by applying the variants (as above) of stimulation protocols.

Models can be, and are, modified relative to the published version; often this can be to correct typographical errors [55] or to test alternative parameterisations. All of which emphasises the absolute requirement to standardise and version control such modifications, so that a simulation run in different labs, by different modellers, can produce mathematically identical results. An equally important aspect of ensuring reliable results is to ensure that simulation codes are well tested or verified 100, 101. To illustrate the issues that can arise, we use the Hund & Rudy 2004 model to demonstrate the importance of parameter control. Protocol-related parameters (number of paces, amplitude and duration of stimulation pulse) were varied to test the impact on the output. To see whether the protocol-related parameters can also influence the cardiac risk prediction, quinidine was taken as a case study, with IC_50_ values extracted from the literature – *I*_Kr_: 1.5 μm [67], *I*_Ks_: 50 μm [102], *I*_Na_: 9.4 μm [67], *I*_CaL_: 15.5 μm [67] – and an AP was simulated with increasing concentration of quinidine (ten logarithmically, equally-spaced concentration steps between 0 and 100 μm), and with varied protocol parameters [number of paces: 1–200 paces; stimulation pulse duration: 3 (original value from the publication), 4 or 5 ms; stimulation pulse amplitude: −15 (original value from the publication), −18, −20, −22 or −25 μA/μF]. From Fig. 5a,b, one can observe that the stimulation pulse duration, amplitude and the number of pulses all influence the prediction. Although in this case the number of pulses has a smaller influence on the outcomes compared with the stimulation pulse amplitude and duration, some other models such as Mahajan model [49] are very sensitive to the number of pulses. In Fig. 5c (ci to ciii: each subfigure shows AP traces at one concentration point of quinidine), the AP traces simulated with 1–200 paces and with different stimulation pulse amplitude and duration are plotted (black); AP traces simulated using original stimulation pulse specification and after 200 paces are plotted in red. APD_90_ and maximal upstroke velocity were calculated from all AP traces simulated (with different stimulation pulse and after different number of paces) and are plotted against quinidine concentration (Fig. 5di,ii, respectively). Increasing variation is seen (Fig. 5c,d) as concentration of quinidine increases.

## Integration of PBPK modelling and cardiac modelling

**Figure 6:**
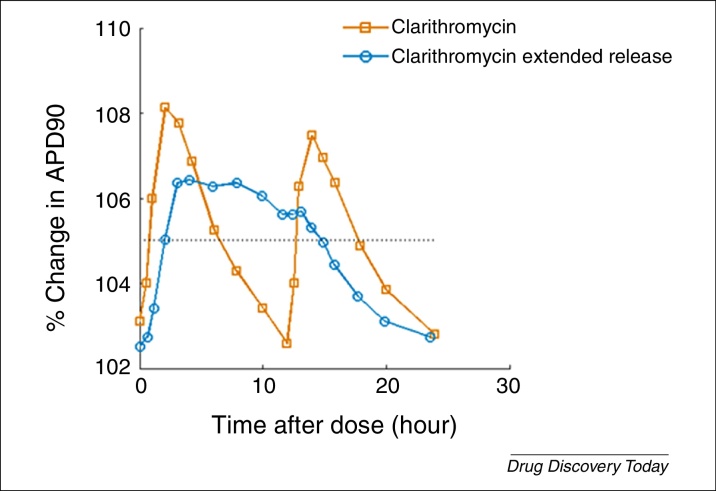
Integrating an understanding of PK allows translation into a cardiac biomarker, in this case changes to action potential duration (APD_50_ and APD_90_) for two formulations of clarithromycin.

Prediction of cardiovascular effects for different formulations and dosing schedules requires a solid understanding of *in vivo* PK in the target patient population, which is where a PK model can prove a valuable tool. In particular, PBPK modelling techniques can also support the prediction of concentration time courses in the tissue of interest, namely the myocardium, and PK drug–drug interactions. A goal in coupling these modelling approaches is to support the link from bench to bedside in a more informative and accurate way. An opportunity exists to collect and collate experimental or observational information from across the continuum (Fig. 3) to instruct the models on how they should be parameterised and assess how they predict (at least for that limited sample). In doing so we can better understand the experimental variability that exists for, for example, patch-clamp data and therefore which value (or range of values) to use in simulations [60]. In Fig. 6 we show an example of linking PK with a cardiac model to simulate two related drug formulations with different clearance rates (one fast and one slow). It exemplifies the importance of applying a QSP approach, which looks to integrate our understanding of drug PK profile over time to these mechanism-based cardiac models. PK profiles of two different forms of clarithromycin were taken from the literature [103]; AP simulation was performed at data points along the PK profiles using the O’Hara–Rudy model [46]. The hERG IC_50_ (∼30 μm) of clarithromycin was taken from the literature [95]. The PK profiles and predicted APD_90_ and APD at 50% repolarisation (APD_50_) are shown in Fig. 6.

## Contribution of *in silico* tools to reduce animal usage

*In silico* methodologies play an important part in the 3Rs: the refinement, reduction and replacement of tests in live animals. The *in silico* approach helps to identify safety liabilities and to remove toxic molecules from the pipeline long before animal experiments are conducted. This allows limiting drug testing to compounds with less toxic effects and higher chances for success. Computer models are, therefore, becoming an important tool for development of alternative testing methods that efficiently integrate complementary information derived from experimental data. The *Toxicology Testing in the 21st Century* document released by the US National Academy of Sciences contains recommendations on how to move the risk identification from descriptive to mechanistically based safety assessment including the use of computer-based technologies for the assessment of toxicities [104]. The FDA's Critical Path Initiative pipeline contains various computational science activities including a project on building an *in silico* tool for screening new drugs for QT prolongation potential, using human clinical trial data [105]. And there are several examples where *in silico* cardiac approaches have reduced animal tests. At AstraZeneca, although extensive screening of all key ventricular ion channels has significantly reduced overall cardiac ion channel liability, the implementation of an *in silico* cardiac model [67] for interpreting those remaining actives meant that an isolated myocyte study could be scaled down (from approximately 23 to one dog per year) via the simulation approach (C. Pollard, *personal communication*). Likewise, at GlaxoSmithKline *in silico* models are helping to profile and select an increased number of compounds while reducing the *ex vivo* rabbit wedge model by approximately 50% (J. Louttit, *personal communication*). Replacement and reduction of animal testing methods in different drug safety areas is not only being used in research but has also been taken up into EU law and the OECD regulations (https://eurl-ecvam.jrc.ec.europa.eu/).

A prerequisite for regulatory acceptance is validation of an alternative method showing that it can provide the same or better protection of human health when compared to traditional methods. Validation includes demonstration of relevance and reliability of a method for a specific purpose and it serves to facilitate and/or accelerate the international (regulatory) acceptance [106]. In general, the following aspects have to be considered when developing alternative methods using a combination of experimental data, computer models and integrative approaches:

• Scientific relevance, characterisation, standardisation and affordability of a biological model.
• The physicochemical diversity in the training and validation sets to cover a variety of mechanisms of action.
• Strategic fit (e.g. prioritisation of approaches for further compound testing in lead identification and optimisation and support in decision making for selected drug candidates at later stages).

To facilitate the acceptance of mechanistically based models for regulatory purposes, new *in silico* tools should be associated with the following information according the OECD principles for QSAR validation 106, 107:

• A defined endpoint.
• An unambiguous algorithm.
• A defined domain of applicability.
• Appropriate measures of goodness of fit, robustness and predictivity.
• A mechanistic interpretation, if possible.

No internationally accepted *in silico* alternative currently exists for a full replacement for all testing of a specific hazard. *In silico* tools alone are not yet sufficiently developed to replace the standard animal tests completely. Therefore, successful development and application of computational tools in drug safety is strongly dependent on their integration and interplay with the experimental approaches. Models are established using either *in vitro* or *in vivo* data or a combination of both to obtain predictions of system-level behaviour. Embracing more *in silico* tools at various levels in our current approaches is a logical consequence of trying to mimic an organ(ism). The higher level of complexity needs to be recapitulated through the use of multiple complementary tests and computational models, which translate the results of scientific research into valid tools that can significantly reduce animal testing while still ensuring the highest level of public health protection.

The practical use of mechanistic mathematical models for safety assessment by regulators is a rather new concept with no specific regulatory guidance in place. Computational safety data are usually submitted on a voluntary basis and are not required. New tools are emerging fast and changing continuously as new data become available – an intrinsic feature of the technology that makes it challenging for potential users to gain sufficient experience. The use of mechanistic models, therefore, must be seen in the context of their potential to model multiple mechanisms of compound toxicity. Customised computational platforms of understandable construction validated with appropriate compound sets provide advantages for practical implementation of *in silico* tools and for successfully integrating *in vitro*, *in silico* and *in vivo* information.

## Concluding remarks

The current paradigm of cardiac safety screening for, primarily, hERG activity to eliminate TdP-causing drugs from reaching the market has reduced incidence of adverse drug-induced TdP. However, this is potentially at the cost of excluding many effective therapies where multichannel effectors might have mitigated hERG-induced cardiac risk. Therefore, the initiative to reconsider the current approach is something to be applauded. However, whereas the aim to reduce the number of incorrectly labelled TdP-liable drugs (false positives) is an important step for accessing more potentially highly efficacious new medicines, it cannot be done (blindly) at the expense of increased false negatives.

*In silico* modelling approaches have great potential to support the drug discovery process through better systematic decision making, animal usage reduction and for providing mechanistic insights as to the compound's intentional (and unintentional) actions. These approaches are already being utilised in the industry and now more mechanistic, systems-pharmacology models are also being investigated for regulatory purposes. However, before wide-scale adoption, careful evaluation and validation need to be conducted on these models, to ensure that we are indeed improving decision making rather than confusing it. It is vitally important to recognise the end user of such tools, so that a consistent interpretation is overlaid to the simulations. For this purpose, development of user-friendly tools and further training of software and model developers and the end user of such simulations to make predictions more readily interpretable is essential for the successful implementation of new initiatives 16, 71, 97, 108. In the consideration of the most appropriate model(s) we advocate a need to consider how the models can respond to future predictions of the unknown compounds rather than simply retrospectively fitting past observations. The reality is probably a need for complementary and fit-for-purpose solution rather than a one-size-fits-all approach, which also aims to support decision making in the understanding of prediction.

Reconciling the demand for ever more detail with the demand that models be sufficiently tractable, mathematically and computationally, to be useful is an essential consideration. These demands could appear to be in opposition to each other. There cannot be any doubt that more detail will be required because there are still many factors that can be involved in arrhythmogenesis that are not yet represented, but could be. These include more detail on metabolic changes underlying forms of arrhythmia and more detail on the feedbacks by which electrical and ionic changes might be involved longer-term in controlling gene expression (electrotranscription coupling). These alternative mechanisms have been attempted elsewhere, for instance inclusion of binding kinetics [52], gene expression [109], safety pharmacology [110], influence of beta-adrenergic activity [111] and cell signalling 112, 113. Because we are already faced with problems of under-determination in the more-complex models, how can these additional demands be compatible with the need for mathematical reduction to simpler models? One aspect is improving the information content of experiments by using models to assist in the experimental design. When we treat experiments as informing us about the parameters in models it becomes possible to optimise the experiments to tell us more about the underlying processes [114]. But there is a paradox: it could be by incorporating more detail that we will eventually find it possible to derive the most useful mathematical reductions. This might be a case of exploring more to focus on less.

For *in silico* cardiac models, many studies and providers have evaluated the value of a different model or software for predicting cardiac proarrhythmic risk. Yet with a different emphasis on each approach and a different set of evaluation drugs for which the approach is scored, an overall assessment of which platform is most appropriate for onward prediction is difficult. In this review we have discussed approaches that have been previously implemented, how each might respond differently dependent upon the circumstances and the consequent need for caution in implementing a solution. With a careful and thorough approach, there is considerable value to be gained from *in silico* approaches, where the primary motivation is not only about mapping out cardiac biology to high accuracy but in providing a method for supporting decision making within drug discovery and development.

## Figures and Tables

**Table 1:** Compounds selected for CiPA proarrhythmia testing, ranked by torsadogenic risk assessment^a^

**High risk**	**Intermediate risk**	**Low risk**
Azimilide	Astemizole	Diltiazem
Bepridil	Chlorpromazine	
dl-Sotalol	Cisapride	Loratadine
Dofetilide	Clarithromycin	Metoprolol
Ibutilide	Clozapine	Mexiletine
Methadone	Domperidone	Nifedipine
Quinidine	Droperidol	Nitrendipine
Vandetanib	Ondansetron	Ranolazine
	Pimozide	Tamoxifen
	Risperidone	Verapamil
	Terfenadine	

a *Source*: Table 1 from https://dx.doi.org/10.1038/nrd.2015.34.

**Table 2:** Differences in predictive power for TQT study results when varying *in silico* models **(a)** and validation data **(b)**

**Concentration range**	**O’Hara**	**TenTusscher-06**	**Grandi**
**(a)**			
At TQT conc.	62%	50%	59%
10-fold TQT conc.	76%	71%	68%
100-fold TQT conc.	88%	79%	71%

**Concentration range**	**Q-Patch**	**Manual and Q-Patch**
**(b)**		
At TQT conc.	62%	71%
10-fold TQT conc.	76%	91%
100-fold TQT conc.	88%	91%

Data adapted, with permission, from [58].

**Table 3:** Comparison of compound assessment from previous *in silico* studies

**Study**	**Compounds in common with at least one other study**^a^	**No. unique*****in silico*****study compounds**	**Compounds common to CiPA list**^b^
**Mirams 2014 (39 cmpds)**	11	28	1
**Kramer 2013 (55 cmpds)**	28	27	20
**Davies 2012 (53 cmpds)**	6	47	5
**Mirams 2011 (31 cmpds)**	18	13	13

a Seven compounds have appeared in more than two studies: amiodarone, cisapride, dofetilide, nifedipine, pimozide, quinidine and terfenadine. No compounds have been used in every study.

b Eight compounds (azimilide, clarithromycin, domperidone, metoprolol, ondansetron, ranolazine, tamoxifen, vandetanib) from the CiPA list have not previously been the subject of an *in silico* cardiac study.

## Glossary

APD_90_: action potential duration at 90% repolarisation (time to return to the resting membrane potential)
Bidomain equations: differential equations representing the time and space changes in charge in two compartments (intra- and extra-cellular), owing to diffusion of charges separately and/or differently in each compartment, and transmembrane currents linking the two
Cardiac arrhythmia: irregular heartbeat
Cardiac action potential: the change in the electrical membrane potential of the cardiomyocyte
CiPA: the comprehensive *in vitro* proarrhythmia assay, a new paradigm for nonclinical assessment of cardiac risk, by a combination of *in vitro*, *in silico* and integrated human-cell-based assay techniques
Ensemble models: an approach that uses several sets of model parameters, rather than a single set, to give a collection of predictions. Generally used to provide an indication of variance in the prediction or to represent a population
hERG: human Ether-a-go-go-related gene encodes the (α-subunit of the) protein underlying *I*_Kr_, a major repolarisation current in the cardiomyocyte during an action potential
Hodgkin–Huxley model: a mathematical method, extended to represent the cardiac ion channel, that uses independent variables to describe the voltage and time-dependent gating properties of an ion channel
*In silico* models: describes models that use computers and algorithm-based approaches for representing a system. Particularly used to contrast to, for example, *in vitro* (cell-based) models or *in vivo* (animal-based) models
Markov state model: an alternative method to Hodgkin–Huxley approaches that represents the open, closing and inactivation of ion channels as a sequence of dependent states and transitions, which can be dependent upon drug binding and ion concentrations, voltage or time
Model calibration: the process by which all or a subset of parameters or components of the model are adjusted or modified to best fit with a set of previously measured outcomes
Model validation: the process of testing the performance of an *in silico* model against a set of measurements that are (usually) not part of the model calibration process
Monodomain equations: differential reaction–diffusion equation representing the time and space changes in charge throughout a single spatial ‘domain’ – the inside of cells – and the contributions that the transmembrane currents make to this
Parameter set: this term is used to describe the set of values that form components of the algorithm behind the *in silico* model that are fit to characterise the system. These parameters might or might not have a direct physical meaning (e.g. the expression level of a protein or the gating kinetics of an ion channel)
PK/PD: the study of how the body responds to a dose of drug [i.e. the drug concentration over time (PK) and the observed effects on the body by the dose (PD)]
Quantitative systems pharmacology (QSP): a discipline that takes a more mechanism-systems-based approach at modelling the pharmacology of a drug and its effects on the body
Top-down, bottom-up, middle-out modelling: these terms are used to describe the starting point for the construction of an *in silico* model to explain the phenomena of interest. Bottom-up describes approaches that start with the individual molecules, whereas top-down begins at the physiological level and attempts to include only the physiological elements necessary for explaining the phenomena. Middle out is a more pragmatic attempt to address the short comings of the top-down and bottom-up approaches
Torsade de Pointes: a polymorphic ventricular arrhythmia that exhibits distinct characteristics on the electrocardiogram (ECG) and can potentially lead to sudden cardiac death
